# A novel TLR-8 agonist attenuates nasal symptoms/congestion in both dog and human allergen challenge studies

**DOI:** 10.1186/1476-9255-10-S1-P14

**Published:** 2013-08-14

**Authors:** Edward G Barrett, Karin Rudolph, Maura Matthews, Greg Dietsch, Robert Hershberg

**Affiliations:** 1 Lovelace Respiratory Research Institute, Albuquerque, NM, 87108, USA; 2 VentiRx Pharmaceuticals, Inc., Seattle, WA, USA

## Rationale

VTX-378/1463 are selective TLR8 agonists that interact with monocytes, macrophages, and myeloid dendritic cells. We hypothesized that delivery of VTX via nasal spray directly to sites of allergic inflammation might initiate rapid anti-allergic activity.

## Methods

(Dog) Ragweed (RW)-sensitized dogs (n=5) were treated with VTX-378 (100, 500 or 1000 μg/dog), given ~ 24 hours prior to RW challenge. Two VTX-378 pretreatments of 250 or 1000 μg/dog, spaced either 3, 4 or 7 days apart were also evaluated. Changes in nasal cavity volumes (congestion) were determined by acoustic rhinometry. (Human) Subsequently, a randomized, double blind, placebo-controlled study was conducted in-season, and enrolled 80 adults with confirmed atopy to grass pollen. Two dosing regimens were compared to placebo: ascending dose (GrpA; 25, 50, 75, 100 μg) and fixed dose (GrpB; 62.5 μg x 4 doses). Subjects were dosed on Days 1, 8, 15, and 22 with VTX-1463. On day 24, subjects underwent grass allergen exposure. The primary endpoints were the average change over 6 hours of allergen exposure in Total Nasal Symptom Score (TNSS; sum of scores for nasal congestion, itching, sneezing and rhinorrhea) and Active Anterior Rhinometry (ARR).

## Results

(Dog) Statistically significant improvements in nasal congestion (44.5% ± 8.7%, 59.1% ± 15.1% and 56.5% ± 10.0% increase in nasal cavity volume *vs* vehicle) were seen at 100, 500 and 1000 μg/dog doses, respectively, following 24 hour pretreatment. Two 250 μg/dog doses at Day -4 and Day -1, resulted in efficacy comparable to a single 500 μg/dog dose given at Day -1 (58.9% ± 10.3% versus 59.1% ± 15.1%). At a dose of 1000 μg/dog, VTX-378, pretreatment on Days -8 and -1, Days -5 and -1 or only Day -1 significantly attenuated nasal congestion (71.9% ± 7.7%, 65.4% ± 10.3% and 56.5% ± 10.0%, respectively). (Human) Significant improvements in TNSS were observed in both GrpA (p=0.008) and GrpB (p=0.012). AAR trended towards a benefit in both groups but did not reach statistical significance.

## Conclusions

Overall, VTX conferred clinical benefit in a dose-dependent manner in both the preclinical dog allergen challenge model and the human allergen challenge chamber studies. These findings suggest that in the context of nasal allergies the dog model can be predictive of dose and clinical efficacy.

